# Real-time augmented feedback for gait training: are gait responses affected by the choice of target parameter?

**DOI:** 10.3389/fbioe.2025.1645390

**Published:** 2025-08-08

**Authors:** Mathilde Legrand, Florette Grenet, Olivia Hochstrasser, Andreas Luft, Roger Gassert, Olivier Lambercy, Chris Easthope Awai

**Affiliations:** ^1^ Rehabilitation Engineering Laboratory, ETH Zurich, Zurich, Switzerland; ^2^ GIPSA-Lab Grenoble Images Parole Signal Automatique, University Grenoble Alpes, Centre National de la Recherche Scientifique (CNRS), Grenoble INP, Grenoble, France; ^3^ Data Analytics & Rehabilitation Technology (DART), Lake Lucerne Institute, Vitznau, Switzerland; ^4^ Cereneo - Center for Neurorehabilitation, Vitznau, Switzerland; ^5^ Vascular Neurology and Neurorehabilitation, Department of Neurology, University Hospital of Zurich, Zurich, Switzerland; ^6^ Future Health Technologies Programme, Singapore - ETH Centre, Campus for Research Excellence and Technological Enterprise (CREATE), Singapore, Singapore; ^7^ The LOOP Zurich – Medical Research Center, Zurich, Switzerland

**Keywords:** augmented feedback, gait symmetry, gait training, motor adaptation, therapy personalization

## Abstract

**Background:**

Augmented visual feedback (AVF) is a promising approach for gait rehabilitation after stroke. However, we still lack crucial knowledge about how to most efficiently use it.

**Research Question:**

How does the selection of the gait parameter targeted by the AVF signal influence the global motor response (i.e., overall gait pattern)?

**Methods:**

24 healthy young participants (mean age 25.3 ± 5.3 years old) performed one session of treadmill walking while receiving real-time AVF driving them towards gait asymmetry. AVF was given during 3 × 10 mins on three different gait parameters: stance time (ST), ankle plantarflexion at toe-off (APL) and push-off force (POF). We analyzed gait responses throughout, with a focus on parameter-specific (local) changes in symmetry ratio, and global gait pattern changes, quantified by correlation coefficient and Gait Deviation Index (GDI).

**Results:**

When using ST and POF feedback targets, participants successfully modified their local asymmetry by an average of 10%. Correlation analysis (Spearman) indicated that the modulated gait propagated across parameters, with a fair correlation (|r|=0.3−0.5) between ST and APL, POF or vertical ground reaction force and between POF and ST, swing time, step length, step height or vertical ground reaction force. However, global gait pattern was only negatively influenced by ST feedback (GDI -7.9 points with p≤0.01 ). Conversely, APL did not lead to significant local symmetry modulation.

**Significance:**

Our results show that the efficacy of AVF is dependent on the selected target parameter. This choice also seems to affect how local symmetry changes affect global motion patterns. This work is a first step towards a more comprehensive understanding of the direct and indirect impact of AVF on gait response, which is crucial before using AVF for clinical applications.

## 1 Introduction

Healthy gait largely contributes to mobility, functional independence and good quality of life (Park and Kim, 2019; Cohen et al., 2018). When gait is impaired after a neurological injury, one major priority is to recover the associated motor functions through rehabilitation. After stroke, for instance, gait impairment can affect between 30% and 50% of patients (Friedman, 1990; Jørgensen et al., 1995; Katan and Luft, 2018; Feigin et al., 2021). Current rehabilitation interventions, such as intensive physical therapy, rely on motor learning and motor control processes (Richards et al., 2015). While these processes are driven by therapists in conventional approaches, through their observations and oral comments, technology-based augmented feedback is a promising method that could complement conventional therapy in a more persistent, objective and precise manner (Schmidt et al., 2018; Sigrist et al., 2013a). In addition to intrinsic feedback (via sensory inferences), augmented feedback can provide information on a physiological or motor performance (e.g., kinematics or electromyography), through an external stimulus (Sigrist et al., 2013a). This stimulus can be shaped in many different ways via the motion parameter to target with the augmented feedback, the modality (visual, haptic and/or auditory), the mapping (e.g., which sound or visualization conveys the information), the duration or the frequency (Sigrist et al., 2013a).

Many studies focused on the impact of augmented feedback on motor learning for healthy participants, looking at the influence of feedback modality, duration, frequency or its interaction with the task difficulty (Sigrist et al., 2013a; Dozza et al., 2006; Sigrist et al., 2013b). The positive conclusions encourage to explore the use of augmented feedback for motor rehabilitation, and in particular for gait rehabilitation, with the hypothesis that feedback may improve or speed up recovery. Several pilot studies thus investigated the motor response of individuals with stroke in reaction to a visual, auditory, or haptic feedback signal (Afzal et al., 2019; Cha et al., 2018; Liu et al., 2020; Genthe et al., 2018; Jung et al., 2020). These studies tend to show the benefits of augmented feedback on gait recovery, calling now for longitudinal studies and high-quality clinical trials to confirm these findings at a bigger scale (Spencer et al., 2021; Angelis et al., 2021). However, while the influence of the modality or the influence of the mapping are often a topic of interest (Afzal et al., 2019; Sigrist et al., 2013b; Kinnaird et al., 2016; Chamorro-Moriana et al., 2018), the crucial role of the selection of the parameter to be targeted by augmented feedback is often neglected. The effects of selecting different target parameters for augmented feedback have so far not been systematically evaluated, despite being a crucial design criteria.

Augmented feedback usually targets one parameter only, while the different gait parameters can be interdependent (Kim and Eng, 2003; Balasubramanian et al., 2007). It is particularly the case when considering gait asymmetry, a common characteristic of post-stroke gait (Patterson et al., 2010; Patterson et al., 2008; Ogihara et al., 2020), which is a product of changes in multiple movement parameters. For instance, previous studies indicate correlations between temporal symmetry and ground reaction force and between spatial symmetry and push-off force symmetry (Kim and Eng, 2003; Balasubramanian et al., 2007). When tackling gait asymmetry with augmented feedback, two important questions arise: (i) does the choice of the target parameter influence the effects of augmented feedback on local modifications, i.e., on the modifications of the target parameter itself? and (ii) does the choice of the target parameter influence the augmented feedback-induced gait pattern modification on a global level?

In this study, involving healthy participants, we compared the impact of augmented visual feedback (AVF) when targeting three different gait parameters individually: stance time, push-off force and ankle plantarflexion. These parameters were selected as they are commonly affected in stroke patients (Olney and Richards, 1996; Li et al., 2018). With a feedback scenario that enhanced asymmetry (i.e., enforced asymmetric gait in healthy subjects) and simulated stroke-like conditions, we analyzed the impact of the AVF through the evolution of each target parameter during 10 min of treadmill walking, and through a comprehensive analysis of the whole gait pattern. We hypothesize that healthy adults would be more responsive to AVF on the spatiotemporal parameter (stance time), adopting a significant asymmetry, since the focus is more external (effect of one’s movement) than for kinetics and kinematics (that are directly one’s own movement) (Magill and Anderson, 2014; Sigrist et al., 2013a; Durham et al., 2009). We also hypothesize that, for each target parameter, the change induced by the AVF would influence other key gait parameters. This work builds on the results of a pilot study that investigated the impact of AVF on stance time on the whole gait (Legrand et al., 2024), where we showed that AVF on temporal symmetry affects both kinetic symmetry and the kinematic pattern of gait. Here we extend this by comparing AVF on stance time with AVF on push-off force and on ankle plantarflexion, with a higher number of participants.

## 2 Materials and methods

### 2.1 Experimental protocol

Twenty-four healthy adults (15 women and 9 men, mean age 25.3 ± 5.3 years old, mean height 172 ± 10 cm, mean weight 68 ± 12 kg), with no history of neurological, orthopedic or rheumatologic disease which affects gait and balance function, participated in the study. The latter was approved by the ETH Zurich ethics committee (No 2022-N-41) and all participants gave their informed consent, in accordance with the Declaration of Helsinki and Good Clinical Practice. The number of participants was based on previous gait feedback studies with healthy participants (Koritnik et al., 2010; van den Noort et al., 2014; Reh et al., 2022; Tomita et al., 2024).

**FIGURE 1:**
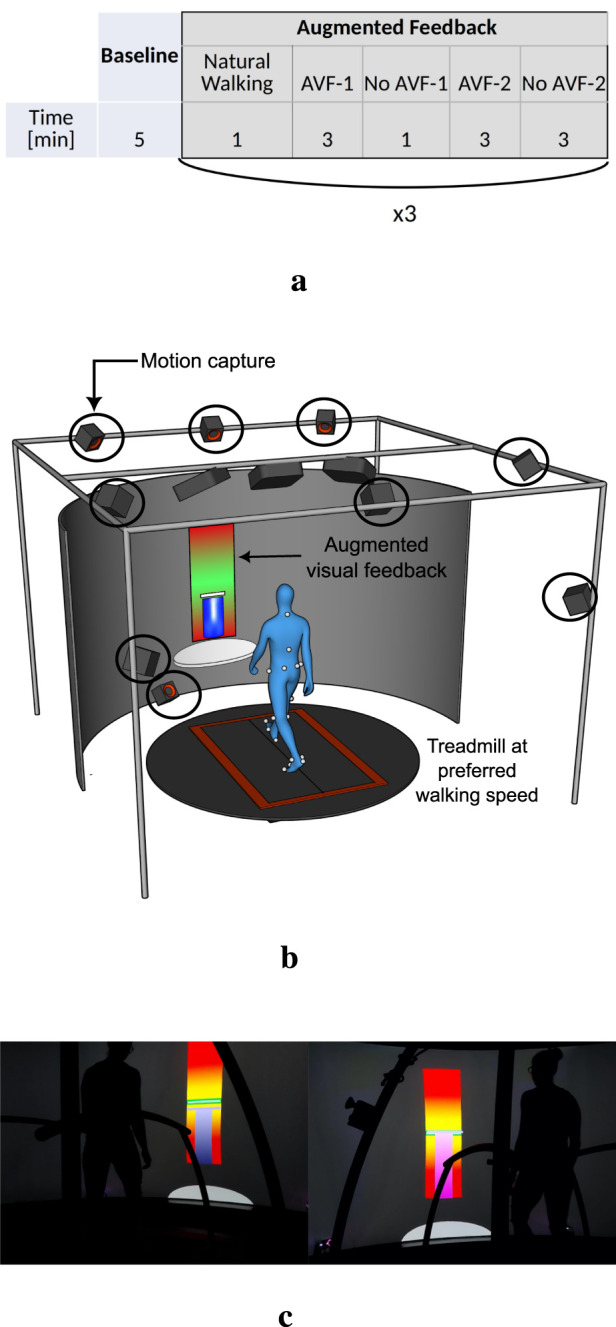
Timing and experimental environment of the treadmill walking session **(a)** Phases of the experiment, with the corresponding duration **(b)** CAREN environment **(c)** Visual AVF.

Each participant performed one session of treadmill walking, composed of a baseline walking followed by walking with AVF. During baseline, participants were asked to walk naturally on the treadmill, at their preferred gait speed, for 5 minutes. This phase allowed participants to get used to treadmill walking and to determine each participant’s preferred gait speed (Meyer et al., 2019). The walking speed was then kept constant during the AVF phase: it was equal to 80% of the preferred gait speed of the participant, to make asymmetry modulation easier to manage. The AVF phase was divided into three conditions, each targeting a different gait parameter: stance time (ST - spatiotemporal), antero-posterior force at toe-off or push-off force (POF - kinetics) and ankle plantarflexion (APL - kinematics). The order of the conditions was randomized between participants. Each condition comprised 1 min of natural walking (NW), followed by two periods of intermittent AVF: (1) 3 mins with AVF (AVF-1), 1 min without (no AVF-1) and (2) 3 mins with AVF (AVF-2) and 3 mins without (no AVF-2, see Figure 1a). During the two periods without AVF, participants were instructed to try to retain the same gait pattern as the one learnt during the periods with feedback. The last “no feedback” period (no AVF-2) lasted 3 mins in order to be able to observe any retention or decay of the learning at the end of the training session. We chose to provide intermittent feedback (i.e., alternating periods with and without AVF) to study retention effects and avoid over-reliance on the visual feedback. A break of few minutes (ended by the participants) was provided between each condition.

The entire experiment was performed in a Computer Assisted Rehabilitation Environment (CAREN, Motek), which includes a dual-belt treadmill with integrated force plates, a motion capture system (Vicon Motion Systems Ltd.), and a 180 ° screen to project virtual environments or any kind of visual display (see Figure 1b). Participants were equipped with optical markers on anatomical landmarks, as defined in the HBM2 model (van den Bogert et al., 2013). Gait parameters of interest (i.e., left and right ST, POF and APL) were processed in real-time with the D-Flow software (Motek Medical B.V.) (Geijtenbeek et al., 2011) and used as inputs for the visual feedback.

### 2.2 Augmented visual feedback

In this study, the objective of the AVF was to induce asymmetric walking in healthy participants. Three different targets were chosen, based on common observations from stroke patients (Olney and Richards, 1996; Li et al., 2018): a shorter ST, a smaller POF or a smaller APL at toe-off, on the left side. With stroke patients, the same type of AVF would be used to improve towards gait symmetry. The characteristics of the visual feedback were inspired from other studies (Genthe et al., 2018; van den Noort et al., 2014; Colborne et al., 1993) and adapted following the outcomes of our pilot study (Legrand et al., 2024). Continuous feedback was selected rather than terminal, since this has shown better efficacy for learning of novel, complex motor tasks (Sigrist et al., 2013a). The AVF was displayed on the 180 ° screen in front of the treadmill. The mapping (i.e., what was projected on the screen) was the same for the three gait parameters: the left target parameter (ST/POF/APL) was continuously rendered as a blue bar. The target value was represented as a light green bar, located in the middle of a green zone, in a red-green-red color gradient, as illustrated in Figure 1c. The limits of the green zone represented ± 5% of error above and below the target. The target value was personalized: it was dynamically computed (i.e., at each time step) to reach 80% of individual baseline symmetry ratio. It was defined as $\alpha_{\text{left}}=0.8\times \delta_{0,\alpha }\times \alpha_{\text{right}}$ with α being ST/POF/APL, $\delta_{0,\alpha }$ the baseline symmetry ratio of each parameter, computed as the mean of the symmetry ratio $\delta_{\alpha }=\frac{\alpha_{\text{left}}}{\alpha_{\text{right}}}$ during baseline walking, and $\alpha_{\text{right}}$ taken as the mean over the last three steps of the right leg value. When the target was reached, the blue bar representing the left parameter became purple. We selected 80% of baseline symmetry as target ratio after preliminary tests with several values and different participants. With a less asymmetric target (90% of baseline symmetry), participants tended to keep a symmetric gait despite the AVF. 80% thus induced important enough gait pattern modifications while staying doable by participants. It has been reported as severe asymmetry ratio for the stroke population (Patterson et al., 2008).

Before starting the visual feedback phase, few explanations were given to the participants: (i) it was specified that the side to modify with the feedback was the left side; (ii) the parameter targeted by the feedback was explained (i.e., what is ST/POF/APL); and (iii) participants were instructed to reach the target in the middle of the green zone. We also instructed the participants to maintain their new walking pattern during the no AVF periods. We, however, did not tell them the gait pattern they should achieve (i.e., no information about symmetry) and did not answer if they asked about their performance during the experiment. We deliberately gave limited and standardized guidelines, to let participants explore by themselves and evaluate the ease of understanding of the AVF.

### 2.3 Data analysis

Three complementary analyses were conducted: (i) evolution of the target symmetry ratio to assess local effects of AVF, (ii) Spearman correlation coefficient between the target parameter and other gait parameters to assess the propagation of the modulated gait across parameters and (iii) Gait Deviation Index (GDI) to assess overall gait pattern changes.

Motion capture data were processed with Vicon ${\text{Nexus}}^{TM}$ (Vicon Motion Systems Ltd. United Kingdom) before being exported and analyzed with the Gaitalytics Python library (2023). Gait events, spatiotemporal parameters and kinetic parameters were computed based on the 3D motion capture data. Kinematic parameters were directly retrieved from the D-Flow software (Motek Medical B.V.). Symmetry ratios δ were computed for a wide range of frequently reported gait parameters: ST, swing time, step length, step width, step height, APL at toe-off, maximum knee flexion, mean vertical ground reaction force (vGRF) during stance phase and POF. They were computed for each gait cycle of the participants.

To evaluate whether the local changes due to AVF depended on the target parameter, we looked at the evolution of the target symmetry ratio along each condition: we looked at $\delta_{ST}$ during ST condition, $\delta_{\mathrm{POF}}$ during POF condition and $\delta_{\mathrm{APL}}$ during APL condition, during the 10 mins of walking with intermittent feedback. For visualization, each phase (NW, AVF and no-AVF) was divided into 10 time points and the symmetry ratios δ were averaged between each time point and then over the participants. Statistical analysis with Linear Mixed Models ( α level of 5%) was conducted (i) considering $\delta_{NW}$ and $\delta_{\mathrm{AV F-2}}$ , with phases as fixed effects, for each condition separately, and (ii) considering $\delta_{NW}-\delta_{\mathrm{AV F-2}}$ of the three conditions, with the conditions as fixed effects. $\delta_{NW}$ was the mean of δ over the NW phase and $\delta_{\mathrm{AV F-2}}$ was the mean of δ over the ten last gait cycles of AVF-2, for each participant individually. We also checked that the order of conditions did not affect the results, comparing $\delta_{\mathrm{AV F-2}}$ for each sub-group (i.e., when each target parameter was tested first, second or third). The reported effect size is the Cohen’s d . For the APL condition, 4 participants had to be excluded due to poor data quality (markers required for APL computation were not visible during the whole condition).

We then analyzed the impact of the AVF on the entire gait with

1. The evolution of $\delta_{ST}$ , $\delta_{\mathrm{POF}}$ and $\delta_{\mathrm{APL}}$ during the three conditions,
2. The Spearman correlation coefficients, between δ of the parameter targeted by the AVF and δ of the other spatiotemporal, kinematic and kinetic gait parameters. The Spearman coefficients were computed within individual participants, using values from all gait cycles of NW and of AVF-2,
3. The change of the scaled (GDI) (Schwartz and Rozumalski, 2008), a multivariate measure of overall gait deficit, between NW and AVF-2.

For the last two analyses, only the AVF-2 phase was evaluated, since participants had the longest time to get used to the feedback and to learn an asymmetric gait pattern. No statistical tests were performed with the Spearman correlation coefficient, due to the non-independence between gait cycles. Wilcoxon tests ( α level of 5%), were performed between NW-GDI and AFV-2-GDI (mean GDI over 10 gait cycles for each participant). Due to missing data (undetected markers), the GDI and APL trajectories could not be calculated for 5 participants in the ST condition and 7 in the POF condition. These participants were still included in analyses 1 and 2, when APL was not required. Statistical analyses were adjusted accordingly.

**GDI computation:** The gait features required for the GDI, as described in (Schwartz and Rozumalski, 2008), must include a large scope of gait patterns, healthy but also pathological. In this study, they were obtained by taking the gait vectors from the stride in the middle of each phase (NW, AVF and no-AVF), for left and right sides, for all participants. The total number of gait vectors used to compute the gait features was thus equal to 1 stride × 2 sides × 3 phases × n participants = 6 × n, with n = [19, 17, 20] for ST, POF and APL respectively. The middle-phase stride was selected in order to remove any transition effects. We checked that the obtained features allowed to reconstruct the gait of the participants. The control dataset included one left and one right strides of NW from all the participants. Its feature components were averaged to describe the average control gait to compare against, as described in (Schwartz and Rozumalski, 2008). Finally, using the gait features and the average control feature components described above, we computed the GDI, averaged over ten gait cycles, (i) during NW and (ii) at the end of AVF-2, when participants were expected to exhibit best overall performance. As explained in (Schwartz and Rozumalski, 2008), scores above 100 represent the absence of gait deficit (i.e., no difference with the control gait, here NW), while a decrease of 10 points represents a shift of one standard deviation away from the control mean. A GDI below 90 can typically be considered indicative of mild gait impairment (Mar et al., 2020).

## 3 Results

### 3.1 Local impact of the augmented visual feedback

**FIGURE 2:**
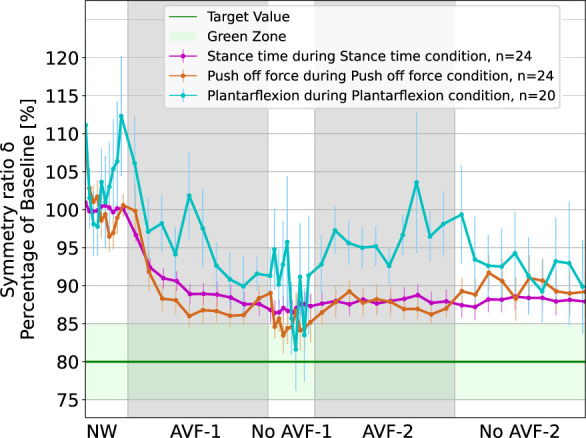
Evolution of symmetry ratios $\delta_{ST}$ , $\delta_{\mathrm{POF}}$ and $\delta_{\mathrm{APL}}$ during AVF on ST, POF and APL respectively. The symmetry ratio was averaged every 10% of each phase for each participant, before taking the mean over all participants. NW: Natural walking. AVF: augmented feedback. n is the number of participants used in the analysis. The error bar is the standard deviation of the mean.

The order of conditions did not affect the results, with no learning or fatigue effect. The evolution of the symmetry ratios, $\delta_{ST}$ , $\delta_{\mathrm{POF}}$ and $\delta_{\mathrm{APL}}$ during their respective feedback condition is plotted in Figure 2. For ST and POF (in purple and orange), at a group level, the participants converged to an asymmetric gait, with δ<90% , few gait cycles after the start of the AVF. They kept this asymmetric walking: δ at the end of AVF-2 is significantly different from δ during NW (p<0.01) , for both ST and POF ( d=2.3 and d=1.7 resp.). Participants also managed to keep the asymmetry even when the AVF was removed (i.e., during the “no AVF” phase). However, the target value of 80% was not achieved. At the personal level, individual analysis showed that 3 of 24 participants did not respond to ST, and 5 of 24 did not respond to POF. Few other participants adopted an asymmetric gait but without reaching the target value (7 for ST condition; 3 for POF condition). 58% of participants reached 80% of asymmetry on ST and 66% reached it for POF.

Regarding the APL condition (in blue), the response is not as clear as for ST and POF. The motor response at group-level was highly variable: the symmetry ratio fluctuated from 100% to 115% during NW and, despite a decrease during AVF-1 with δ close to 90%, the symmetry ratio kept fluctuating during the rest of the condition. We could also observe a high standard deviation of the mean, showing an important inter-subject variability. No significant effect of AVF was visible, with no significant change between NW and AVF-2 (p=0.87) . The effect size was very small (d=0.04) due to the symmetry ratio fluctuations and the high inter-subject variability. At the individual level, none of the participants managed to reach and maintain an asymmetric APL with the AVF. When comparing the asymmetry obtained at the end of AVF-2 for each condition, we saw no statistical difference between $\delta_{ST}$ and $\delta_{\mathrm{POF}}$ (p=0.80) , while there was a significant difference between $\delta_{\mathrm{APL}}$ and $\delta_{ST}$ (p=0.03) and between $\delta_{\mathrm{APL}}$ and $\delta_{\mathrm{POF}}$ (p=0.02) .

### 3.2 Propagation of asymmetry across gait parameters

**FIGURE 3:**
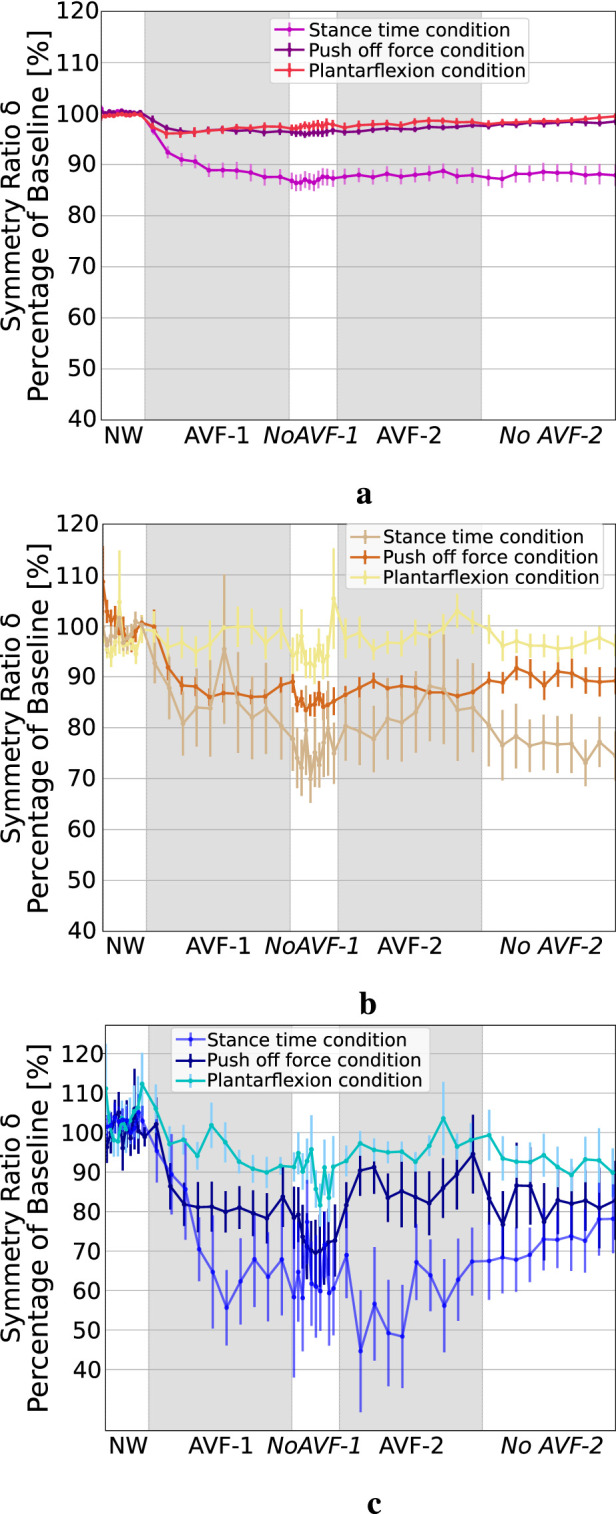
Symmetry ratio of ST, POF and APL during the three AVF conditions. **(a)** ST symmetry ratio. **(b)** POF symmetry ratio. **(c)** APL symmetry ratio.

Figure 3 shows $\delta_{ST}$ , $\delta_{\mathrm{POF}}$ and $\delta_{\mathrm{APL}}$ for the three conditions. We can see that AVF on ST (ST condition) led to important modifications on POF and APL as well, with higher POF and APL asymmetry induced than during the condition where they were targeted by the AVF. $\delta_{\mathrm{POF}}$ reached around 80% during ST condition versus 88% during the POF condition (see Figure 3b) and $\delta_{\mathrm{APL}}$ reached around 60% versus 95% during the APL condition (see Figure 3c). AVF on POF also induced more asymmetric APL than feedback on APL (around 85% versus 95%) but did not induce ST modifications (see Figure 3a). AVF on APL (APL condition) did not lead to ST or POF asymmetry.

In addition to the three parameters targeted by the AVF, we analyzed how AVF impacted the symmetry of other relevant and commonly reported gait parameters. Table 1 gathers the Spearman correlation coefficients between the symmetry ratio of the parameter targeted by the feedback and the other ones. The ranking fair (|r|=0.3−0.5) , moderate (|r|=0.6−0.7) and very strong (|r|=0.8−0.9) is defined according to (Akoglu, 2018; Chan, 2003). Swing time symmetry ratio was very strongly negatively correlated to ST (−0.86). We could also observe fair (close to moderate) positive correlation of $\delta_{ST}$ with $\delta_{\mathrm{APL}}$ , $\delta_{\mathrm{vGRF}}$ and $\delta_{\mathrm{POF}}$ , reflecting the findings shown in Figure 3. $\delta_{\mathrm{POF}}$ had a fair correlation with $\delta_{ST}$ , $\delta_{swing\;time}$ , $\delta_{step\;length}$ , $\delta_{step\;height}$ and $\delta_{\mathrm{vGRF}}$ while $\delta_{\mathrm{APL}}$ had a fair correlation with $\delta_{step\;height}$ only.

**TABLE 1:** Spearman correlation coefficients between the symmetry ratio of the parameter targeted by the AVF and the one of the other gait parameters. Highlighted in yellow are the correlations defined as fair ( |r|=0.3−0.5 ) and in orange the correlations defined as very strong ( |r|=0.8−0.9 ) (Akoglu, 2018; Chan, 2003).

	ST	Swing time	Step length	Step width	Step height	APL at toe-off	Maximum knee flexion	vGRF during stance phase	POF
ST condition	-	−0.86	−0.24	−0.08	0.23	0.51	0.10	0.58	0.56
POF condition	0.37	−0.36	−0.31	0.04	0.33	0.21	0.1	0.37	-
APL condition	0.19	−0.23	−0.14	−0.03	0.47	-	0.09	0.08	0.18

### 3.3 Impact of the augmented visual feedback on the Gait Deviation Index

**FIGURE 4:**
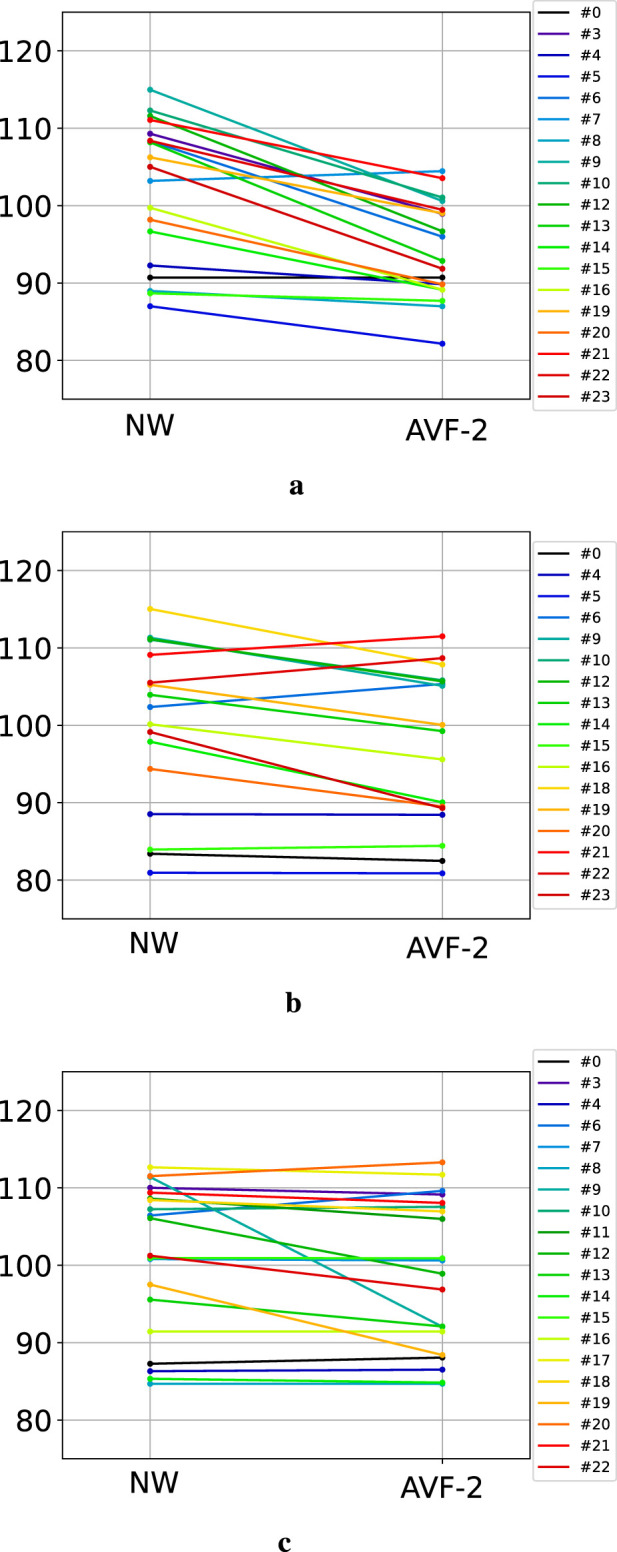
GDI evolution between natural walking and the end of AVF-2, the last feedback phase. Each color represents one participant. **(a)** GDI during ST condition. **(b)** GDI during POF condition. **(c)** GDI during APL condition.

The scaled GDI, calculated for NW and for AVF-2 phases for each participant, are presented Figure 4. During the ST condition, all but one participant decreased their GDI when walking with the AVF (Figure 4a). The GDI decreased by 7.9±5.1 points on average between NW and AVF-2. The GDI of NW and the GDI of AVF-2 were significantly different (p≤0.01) , showing that the shift in ST symmetry had a quantifiable global effect on gait. However, only 7 participants reached a GDI lower than 90 points, indicative of mild gait impairment (Mar et al., 2020). In the POF condition, GDI was reduced by a mean of 3.1 ± 3.9 points, indicating that global motion patterns were less affected by the changes in local symmetry, with only 10 participants (out of 17) presenting a decrease in GDI when walking with AVF (Figure 4b). The only participants showing a GDI < 90 during AVF-2 had a similar GDI already during NW. The difference between GDI during NW and GDI during AVF-2 was still significant (p≤0.05) . In the APF condition (Figure 4c), GDI decreased by only 2.3 ± 4.8 points, indicating minimal impact on the global motion pattern, with no significant difference between GDI of NW and GDI of AVF-2 (p>0.05) . Only three participants (out of 20) showed a drop of more than 5 points between NW and AVF-2 (see #9 , #12 and #19 on Figure 4c). This further corroborates the previous results in which APF demonstrated significant lower performance in creating asymmetric walking patterns.

## 4 Discussion

The objective of this study was to investigate the local and global motor responses to real-time AVF, and to quantify these across different target parameters. With healthy adults, targeting different parameters with visual feedback led to significantly different local motor response patterns, which in turn differentially affected global motion. The AVF drove participants towards gait asymmetry on three separate gait parameters: ST, POF and APL. Results were mixed for APL: participants achieved 5% of asymmetry, which can already be considered as pathological (Patterson et al., 2010), but with large fluctuations, a lot of variability between participants and no significant difference between the last feedback phase and natural walking. This differs with (Colborne et al., 1993), where Colborne et al. presented an AVF targeting ankle kinematics for stroke patients, that had a significant local and global effects (e.g., stride time, knee kinematics) on gait. The AVF design was close to the one of this study (targets to reach at a specific time) but several reasons could explain the distinct results: in Colborne et al. (1993), information on both dorsi and plantarflexion was provided and the AVF was supported by verbal feedback and coupled with auditory feedback (it has been shown that multi-modal feedback can have a higher impact on motor response (Sigrist et al., 2013a)). For ST and POF, participants managed to quickly reach more than 10% of asymmetry and to retain the newly learned gait pattern without the AVF. It thus seems easier to modify gait symmetry when the AVF targets a spatiotemporal or a kinetic parameter. This is aligned with van den Noort et al., who showed that, with healthy subjects, AVF worked better when targeting a kinetic than when targeting a kinematic parameter (van den Noort et al., 2014). With ST or POF, participants focus on the endpoint motion (i.e., the foot), as opposed to the individual joint motion patterns of APL. It may be easier to find how to modify the gait pattern in the first case, compared to when the focus is on one joint. The gait parameter targeted by AVF, with a given mapping, is thus of crucial importance and influences the impact of the AVF on the motor response. Nevertheless, while ST is a discrete parameter and a simple duration to wait until lifting the foot, APL and POF are continuous measures and depend on a whole trajectory. With APL and POF, subjects must be attentive to both amplitude and time and have to find the full trajectory that can lead to the toe-off value targeted by the AVF. The AVF design may not generalize well across the different parameter types, and may require the development of bespoke AVF. Conversely, bespoke AVF would have added a confounder to the experimental design of our study, making it difficult to disentangle the effect of parameter choice from the feedback design. Providing AVF on ST also favors an external focus of attention (i.e., a focus on the effects of one’s movement) compared to providing AVF on POF or APL, which are internal components of one’s movement. Several previous studies showed that augmented feedback with an external focus of attention gave better results than augmented feedback with an internal focus (Magill and Anderson, 2014; Sigrist et al., 2013a; Shafizadeh et al., 2013), which could explain our result on the larger local effect of AVF on ST.

Our work also includes a comprehensive analysis, looking at the impact of the AVF on other components of gait symmetry. We could see that AVF on ST symmetry not only induced local motor adaptations (modifications of $\delta_{ST}$ ), but that these also propagated over other gait metrics to affect the global gait pattern. $\delta_{\mathrm{POF}}$ and $\delta_{\mathrm{APL}}$ were higher during the ST condition than with their respective AVF, $\delta_{ST}$ showed close-to-moderate correlation with $\delta_{\mathrm{APL}}$ and forces symmetry ratio (POF and vGRF), and the GDI decreased to approach a pathological score (below 90) when walking with the AVF. The spatial parameters and the knee flexion, however, were not impacted. Apart from the non-correlation with the knee flexion, this is aligned with our previous results with another group of participants on AVF for ST symmetry (Legrand et al., 2024). Providing AVF for ST thus affected both local and global aspects of gait, while ST symmetry seemed ingrained when AVF targeted another parameter, probably for stability or comfort (see Figure 3a). Regarding the POF condition, AVF generated asymmetry in POF and the induced gait alteration had an impact on $\delta_{\mathrm{APL}}$ (between 80% and 90% of asymmetry). There was also a fair correlation between $\delta_{\mathrm{POF}}$ and the symmetry ratio of spatiotemporal parameters and of vGRF. However, the GDI indicated small modification on global motion patterns. We see two factors that could explain this discrepancy between kinematic effects of AVF on POF measured with APL symmetry ratio and measured with the GDI: (i) APL was affected but not the other kinematic parameters, reducing the effect on the GDI, (ii) the number of participants available to derive the orthonormal basis from which the GDI is computed was low (24 participants), which could bias the results. The second point could also explain why some participants had a GDI lower than 90 points already during NW, while a score around 100 would be expected. Complementary metrics that do not only consider kinematic aspects, such as the GDI-kinetic (Rozumalski and Schwartz, 2011), could also bring a more comprehensive view on global effects. Finally, AVF on APL did not impact the entire gait pattern: neither $\delta_{ST}$ nor $\delta_{\mathrm{POF}}$ was modified during the APL condition, only the step height symmetry ratio had a fair correlation with $\delta_{\mathrm{APL}}$ and the GDI stayed stable between NW and AVF phases. Since the effect of AVF was mild on $\delta_{\mathrm{APL}}$ , the light impact on the entire gait pattern is not surprising. For ST and POF, for which the AVF led to an important asymmetry, we can conclude that AVF on one single gait parameter does not only impact this target parameter but also other characteristics of gait. This global effect can be beneficial as we can treat gait symmetry with a simple AVF on one single parameter. There is thus no need to overload the information included in the feedback signal. Nevertheless, it can also be a drawback: the alteration of non-targeted parameters can be due to compensatory mechanisms, which need to be avoided if they lead to musculo-skeletal disorders. Applied to post-stroke rehabilitation, this means that the effect of the AVF needs to be carefully examined depending on the subject’s impairment and therapy objectives, calling for personalization.

This study highlights that AVF on gait symmetry induces gait changes that exceed the target parameter and that these changes depend on the latter. In this work, we used the symmetry ratio to measure gait symmetry, which is one of the most basic metrics. It is widely used for spatiotemporal parameters (Viteckova et al., 2018; Patterson et al., 2010) but is maybe not the most adequate for kinetics and kinematics, due to the range of their values. Metrics such as the Robinson index (Robinson et al., 1987) or the symmetry angle (Zifchock et al., 2008) could be more suitable, since they were specifically defined for kinetic and kinematic variables and have less dependence on small values. Moreover, we explored one type of AVF only (continuous and visual) and it is still to be explored whether our results are still valid with other modalities (haptic, auditory) or other characteristics (e.g., terminal feedback). The symmetry ratio was encoded in one visualization with one single bar, to keep the AVF signal simple. We yet observed that some participants who did not reach the target symmetry ratio indeed modified their gait but on their left and right sides simultaneously. AVF with more explicit information (left and right sides separately for instance) could have led to more efficient motor response with important gait alteration. Leg dominance may also influence the participant’s response and could be a factor to examine in future works. Finally, further studies should be conducted, first to investigate if the impact of the AVF depends on each parameter individually or if there is a common tendency for the parameters of a same group (spatiotemporal, kinetics and kinematics); second, to evaluate whether results from healthy participants driven towards asymmetry can be transposed to patients driven towards symmetry.

## 5 Conclusion

This study investigated the role played by the target gait parameter on the local and global impacts of AVF. 24 healthy young adults participated in one session of treadmill walking during which they were driven towards gait asymmetry via real-time AVF. This AVF successively gave information on the symmetry ratio of three gait parameters: ST, POF and APL. We showed (i) that the motor response induced by the AVF depended on the target parameter, with a successful lasting alteration for ST and POF and a less clear effect for APL, and (ii) that the successful alterations of ST and POF also resulted in global gait pattern modifications, with distinct effects depending on the target parameter. AVF on ST impacted POF, APL and vertical ground reaction force symmetry, and disturbed the entire gait as illustrated by a significant decrease of GDI. AVF on POF impacted APL, swing time, step length, step height and vertical ground reaction force. Decrease in GDI due to POF asymmetry was less important than the one due to ST asymmetry but still significant. This work highlights the importance of a careful choice of the target parameter used for the AVF as well as the importance of a comprehensive evaluation of the gait patterns when studying the effect of AVF on gait symmetry. A deeper understanding of the indirect impact of AVF on non-targeted gait parameters is crucial to design personalized feedback scenarios, that target an adequate parameter while minimizing compensatory movements.

## Data Availability

The raw data supporting the conclusions of this article will be made available by the authors, without undue reservation.

## References

[B1] Afzal M. R. Lee H. Eizad A. Lee C. H. Oh M. K. Yoon J. (2019). Effects of vibrotactile biofeedback coding schemes on gait symmetry training of individuals with stroke. IEEE Trans. Neural Syst. Rehabilitation Eng. 27, 1617–1625. 10.1109/tnsre.2019.2924682 31247557

[B2] Akoglu H. (2018). User’s guide to correlation coefficients. Turkish J. Emerg. Med. 18, 91–93. 10.1016/j.tjem.2018.08.001 PMC6107969 30191186

[B3] Angelis S. D. Princi A. A. Farra F. D. Morone G. Caltagirone C. Tramontano M. (2021). Vibrotactile-based rehabilitation on balance and gait in patients with neurological diseases: a systematic review and metanalysis. Brain Sci. 11, 518. 10.3390/brainsci11040518 33921655 PMC8072538

[B4] Balasubramanian C. K. Bowden M. G. Neptune R. R. Kautz S. A. (2007). Relationship between step length asymmetry and walking performance in subjects with chronic hemiparesis. Archives Phys. Med. Rehabilitation 88, 43–49. 10.1016/j.apmr.2006.10.004 17207674

[B5] Cha Y.-J. Kim J.-D. Choi Y.-R. Kim N.-H. Son S.-M. (2018). Effects of gait training with auditory feedback on walking and balancing ability in adults after hemiplegic stroke: a preliminary, randomized, controlled study. Int. J. Rehabilitation Res. 41, 239–243. 10.1097/mrr.0000000000000295 29782407

[B6] Chamorro-Moriana G. Moreno A. J. Sevillano J. L. (2018). Technology-based feedback and its efficacy in improving gait parameters in patients with abnormal gait: a systematic review. Sensors 18, 142. 10.3390/s18010142 29316645 PMC5795813

[B7] Chan Y. H. (2003). Biostatistics 104: correlational analysis. Singap. Med. J. 44, 614–619. 14770254

[B8] Cohen J. W. Ivanova T. D. Brouwer B. Miller K. J. Bryant D. Garland S. J. (2018). Do performance measures of strength, balance, and mobility predict quality of life and community reintegration after stroke? Archives Phys. Med. Rehabilitation 99, 713–719. 10.1016/j.apmr.2017.12.007 29317222

[B9] Colborne G. Olney S. J. Griffin M. P. (1993). Feedback of ankle joint angle and soleus electromyography in the rehabilitation of hemiplegic gait. Archives Phys. Med. Rehabilitation 74, 1100–1106. 10.1016/0003-9993(93)90069-m 8215864

[B10] Dozza M. Chiari L. Hlavacka F. Cappello A. Horak F. B. (2006). Effects of linear *versus* sigmoid coding of visual or audio biofeedback for the control of upright stance. IEEE Trans. Neural Syst. Rehabilitation Eng. 14, 505–512. 10.1109/tnsre.2006.886732 17190042

[B11] Durham K. Vliet P. M. V. Badger F. Sackley C. (2009). Use of information feedback and attentional focus of feedback in treating the person with a hemiplegic arm. Physiother. Res. Int. 14, 77–90. 10.1002/pri.431 19107706

[B12] Feigin V. L. Stark B. A. Johnson C. O. Roth G. A. Bisignano C. Abady G. G. (2021). Global, regional, and national burden of stroke and its risk factors, 1990–2019: a systematic analysis for the global burden of disease study 2019. Lancet Neurology 20, 795–820. 10.1016/s1474-4422(21)00252-0 34487721 PMC8443449

[B13] Friedman P. J. (1990). Gait recovery after hemiplegic stroke. Int. Disabil. Stud. 12, 119–122. 10.3109/03790799009166265 2096120

[B14] Geijtenbeek T. Steenbrink F. Otten B. Even-Zohar O. (2011). “D-flow: immersive virtual reality and real-time feedback for rehabilitation,” in Proceedings of the 10th International Conference on Virtual Reality Continuum and Its Applications in Industry, Hong Kong, China, December 11 - 12, 2011 (ACM). 10.1080/10749357.2018.1436384

[B15] Genthe K. Schenck C. Eicholtz S. Zajac-Cox L. Wolf S. Kesar T. M. (2018). Effects of real-time gait biofeedback on paretic propulsion and gait biomechanics in individuals post-stroke. Top. Stroke Rehabilitation 25, 186–193. 10.1080/10749357.2018.1436384 PMC5901660 29457532

[B16] Jørgensen H. S. Nakayama H. Raaschou H. O. Olsen T. S. (1995). Recovery of walking function in stroke patients: the copenhagen stroke study. Archives Phys. Med. Rehabilitation 76, 27–32. 10.1016/S0003-9993(95)80038-7 7811170

[B17] Jung K.-S. Bang H. In T.-S. Cho H.-Y. (2020). Gait training with auditory feedback improves trunk control, muscle activation and dynamic balance in patients with hemiparetic stroke: a randomized controlled pilot study. J. Back Musculoskelet. Rehabilitation 33, 1–6. 10.3233/bmr-170852 31594193

[B18] Katan M. Luft A. (2018). Global burden of stroke. Seminars Neurology 38, 208–211. 10.1055/s-0038-1649503 29791947

[B19] Kim C. Eng J. J. (2003). Symmetry in vertical ground reaction force is accompanied by symmetry in temporal but not distance variables of gait in persons with stroke. Gait and Posture 18, 23–28. 10.1016/s0966-6362(02)00122-4 12855297

[B20] Kinnaird C. Lee J. Carender W. J. Kabeto M. Martin B. Sienko K. H. (2016). The effects of attractive vs. repulsive instructional cuing on balance performance. J. NeuroEngineering Rehabilitation 13, 29. 10.1186/s12984-016-0131-z PMC4793655 26983996

[B21] Koritnik T. Koenig A. Bajd T. Riener R. Munih M. (2010). Comparison of visual and haptic feedback during training of lower extremities. Gait and Posture 32, 540–546. 10.1016/j.gaitpost.2010.07.017 20727763

[B22] Legrand M. L. Magrini C. Branscheidt M. Luft A. Gassert R. Lambercy O. (2024). “Augmented feedback for gait symmetry: a comprehensive evaluation of gait modification,” in 2024 IEEE international conference on biomedical robotics and biomechatronics (BioRob). 10.1109/BioRob60516.2024.10719727

[B23] Li S. Francisco G. E. Zhou P. (2018). Post-stroke hemiplegic gait: new perspective and insights. Front. Physiology 9, 1021. 10.3389/fphys.2018.01021 PMC6088193 30127749

[B24] Liu L. Y. Sangani S. Patterson K. K. Fung J. Lamontagne A. (2020). Real-time avatar-based feedback to enhance the symmetry of spatiotemporal parameters after stroke: instantaneous effects of different avatar views. IEEE Trans. Neural Syst. Rehabilitation Eng. 28, 878–887. 10.1109/tnsre.2020.2979830 32167900

[B25] Magill R. A. Anderson D. (2014). Motor learning and control: concepts and applications. 10th edn. New York: McGraw Hill, Connect Learn Suceed.

[B26] Mar D. Lieberman I. Haddas R. (2020). The gait deviation index as an indicator of gait abnormality among degenerative spinal pathologies. Eur. Spine J. 29, 2591–2599. 10.1007/s00586-019-06252-2 31838597

[B27] Meyer C. Killeen T. Easthope C. S. Curt A. Bolliger M. Linnebank M. (2019). Familiarization with treadmill walking: how much is enough? Sci. Rep. 9, 5232. 10.1038/s41598-019-41721-0 30914746 PMC6435738

[B28] Ogihara H. Tsushima E. Kamo T. Sato T. Matsushima A. Niioka Y. (2020). Kinematic gait asymmetry assessment using joint angle data in patients with chronic Stroke—A normalized cross-correlation approach. Gait and Posture 80, 168–173. 10.1016/j.gaitpost.2020.05.042 32521470

[B29] Olney S. J. Richards C. (1996). Hemiparetic gait following stroke. Part I: characteristics. Gait and Posture 4, 136–148. 10.1016/0966-6362(96)01063-6

[B30] Park J. Kim T.-H. (2019). The effects of balance and gait function on quality of life of stroke patients. NeuroRehabilitation 44, 37–41. 10.3233/NRE-182467 30741699

[B31] Patterson K. K. Parafianowicz I. Danells C. J. Closson V. Verrier M. C. Staines W. R. (2008). Gait asymmetry in community-ambulating stroke survivors. Archives Phys. Med. Rehabilitation 89, 304–310. 10.1016/j.apmr.2007.08.142 18226655

[B32] Patterson K. K. Gage W. H. Brooks D. Black S. E. McIlroy W. E. (2010). Evaluation of gait symmetry after stroke: a comparison of current methods and recommendations for standardization. Gait and Posture 31, 241–246. 10.1016/j.gaitpost.2009.10.014 19932621

[B33] Python library (2023). Gaitalytics. Available online at: https://libraries.io/pypi/gaitalytics.

[B34] Reh J. Schmitz G. Hwang T.-H. Effenberg A. O. (2022). Loudness affects motion: asymmetric volume of auditory feedback results in asymmetric gait in healthy young adults. BMC Musculoskelet. Disord. 23, 586. 10.1186/s12891-022-05503-6 35715757 PMC9206330

[B35] Richards C. L. Malouin F. Nadeau S. (2015). “Stroke rehabilitation,” in Sensorimotor rehabilitation - at the crossroads of basic and clinical sciences (Elsevier), 253–280. 10.1016/S0079-6123(15)00053-9 25890149

[B36] Robinson R. Herzog W. Nigg B. M. (1987). Use of force platform variables to quantify the effects of chiropractic manipulation on gait symmetry. J. Manip. physiological Ther. 10, 172–176. 2958572

[B37] Rozumalski A. Schwartz M. H. (2011). The GDI-Kinetic: a new index for quantifying kinetic deviations from normal gait. Gait and Posture 33, 730–732. 10.1016/j.gaitpost.2011.02.014 21454078

[B38] Schmidt R. A. Zelaznik H. N. Winstein C. Wulf G. Lee T. D. (2018). Motor control and learning. Champaign, IL: Human Kinetics Publishers.

[B39] Schwartz M. H. Rozumalski A. (2008). The gait deviation index: a new comprehensive index of gait pathology. Gait and Posture 28, 351–357. 10.1016/j.gaitpost.2008.05.001 18565753

[B40] Shafizadeh M. Platt G. K. Mohammadi B. (2013). Effects of different focus of attention rehabilitative training on gait performance in multiple sclerosis patients. J. Bodyw. Mov. Ther. 17, 28–34. 10.1016/j.jbmt.2012.04.005 23294680

[B41] Sigrist R. Rauter G. Riener R. Wolf P. (2013a). Augmented visual, auditory, haptic, and multimodal feedback in motor learning: a review. Psychonomic Bull. and Rev. 20, 21–53. 10.3758/s13423-012-0333-8 23132605

[B42] Sigrist R. Rauter G. Riener R. Wolf P. (2013b). Terminal feedback outperforms concurrent visual, auditory, and haptic feedback in learning a complex rowing-type task. J. Mot. Behav. 45, 455–472. 10.1080/00222895.2013.826169 24006910

[B43] Spencer J. Wolf S. L. Kesar T. M. (2021). Biofeedback for post-stroke gait retraining: a review of current evidence and future research directions in the context of emerging technologies. Front. Neurology 12, 637199. 10.3389/fneur.2021.637199 PMC8042129 33859607

[B44] Tomita Y. Sekiguchi Y. Mayo N. E. (2024). Efficacy of a single-bout of auditory feedback training on gait performance and kinematics in healthy young adults. Sensors 24, 3206. 10.3390/s24103206 38794060 PMC11125153

[B45] van den Bogert A. J. Geijtenbeek T. Even-Zohar O. Steenbrink F. Hardin E. C. (2013). A real-time system for biomechanical analysis of human movement and muscle function. Med. and Biol. Eng. and Comput. 51, 1069–1077. 10.1007/s11517-013-1076-z 23884905 PMC3751375

[B46] van den Noort J. C. Steenbrink F. Roeles S. Harlaar J. (2014). Real-time visual feedback for gait retraining: toward application in knee osteoarthritis. Med. and Biol. Eng. and Comput. 53, 275–286. 10.1007/s11517-014-1233-z 25480419

[B47] Viteckova S. Kutilek P. Svoboda Z. Krupicka R. Kauler J. Szabo Z. (2018). Gait symmetry measures: a review of current and prospective methods. Biomed. Signal Process. Control 42, 89–100. 10.1016/j.bspc.2018.01.013

[B48] Zifchock R. A. Davis I. Higginson J. Royer T. (2008). The symmetry angle: a novel, robust method of quantifying asymmetry. Gait and Posture 27, 622–627. 10.1016/j.gaitpost.2007.08.006 17913499

